# Continual learning framework for a multicenter study with an application to electrocardiogram

**DOI:** 10.1186/s12911-024-02464-9

**Published:** 2024-03-06

**Authors:** Junmo Kim, Min Hyuk Lim, Kwangsoo Kim, Hyung-Jin Yoon

**Affiliations:** 1 Interdisciplinary Program in Bioengineering, Seoul National University, Seoul, Republic of Korea; 2 Transdisciplinary Department of Medicine and Advanced Technology, Seoul National University Hospital, Seoul, Republic of Korea; 3 Graduate School of Health Science and Technology, Ulsan National Institute of Science and Technology, Ulsan, Republic of Korea; 4 Department of Medicine, College of Medicine, Seoul National University, Seoul, Republic of Korea; 5 Medical Bigdata Research Center, Seoul National University College of Medicine, 101, Daehak-ro, Jongno-gu, Seoul, Republic of Korea

**Keywords:** Multicenter study, Deep learning, Continual learning, Electrocardiogram

## Abstract

Deep learning has been increasingly utilized in the medical field and achieved many goals. Since the size of data dominates the performance of deep learning, several medical institutions are conducting joint research to obtain as much data as possible. However, sharing data is usually prohibited owing to the risk of privacy invasion. Federated learning is a reasonable idea to train distributed multicenter data without direct access; however, a central server to merge and distribute models is needed, which is expensive and hardly approved due to various legal regulations. This paper proposes a continual learning framework for a multicenter study, which does not require a central server and can prevent catastrophic forgetting of previously trained knowledge. The proposed framework contains the continual learning method selection process, assuming that a single method is not omnipotent for all involved datasets in a real-world setting and that there could be a proper method to be selected for specific data. We utilized the fake data based on a generative adversarial network to evaluate methods prospectively, not ex post facto. We used four independent electrocardiogram datasets for a multicenter study and trained the arrhythmia detection model. Our proposed framework was evaluated against supervised and federated learning methods, as well as finetuning approaches that do not include any regulation to preserve previous knowledge. Even without a central server and access to the past data, our framework achieved stable performance (AUROC 0.897) across all involved datasets, achieving comparable performance to federated learning (AUROC 0.901).

**Supplementary Information:**

The online version contains supplementary material available at 10.1186/s12911-024-02464-9.

## Introduction

With the importance of the size of available data in the deep learning process, multicenter study is one of the most common approaches in studies using medical data. However, using personal information in medical institutions is mainly prohibited, and obtaining permission to access data requires several arduous procedures, such as approval of the institutional review board evaluating the potential risk of using data. Even though access to data is approved, sharing or taking out data is mostly precluded, and additional approval is required according to the bylaws of each institution. Thus, merging and training all involved data at once is practically difficult.

Federated learning is one of the alternatives for decentralized data [1–4]. In federated learning, models are distributed to be trained, aggregated periodically, and distributed again [5]. Federated learning only shares the weights of the trained model without direct access to data, alleviating the potential risk of privacy invasion caused by data sharing. Nevertheless, federated learning requires a central server which is challenging to construct since there are various legal regulations regarding the server containing several institutions [6–9]. Assuming the environment without a central server, executing federated learning is infeasible because automatically merging and distributing trained models until they converge are not available.

As an alternative to federated learning, we propose a continual learning framework for a multicenter study. The goal of continual learning is gradually extending acquired knowledge without catastrophic forgetting [10, 11]. Although continual learning intrinsically focuses on the problems with the sequential stream of data, we reduced the given distributed environment of a multicenter study to the sequential tasks; the data from each institution is provided one by one, and the knowledge from the preceding trained model is retained by suppressing catastrophic forgetting. In this way, the model is trained to fit all involved datasets. Continual learning can be executed without a central server and requires less communication compared to federated learning which needs a central server and communication until the model reaches a convergence state which is unclear when to reach.

Several magnificent continual learning methods have been developed; however, a major challenge in applying these methods to a multicenter study is selecting the most proper one for the specific and inaccessible preceding dataset. To our best knowledge, most studies proposing their continual learning methods evaluated the performance retrospectively. In other words, those studies compared their methods to the baselines using all datasets after all experiments were finished. However, access to only the current institution’s dataset is possible in a real-world setting, and it may be necessary to use a particular method that is suitable for the specific data, rather than relying on the state-of-the-art method.

Inspired by this issue, we focus on selecting a proper continual learning method for each institution in a multicenter study. All involved institutions are assumed to prohibit data sharing strictly and only allow sharing parameters of the trained model. Under this circumstance, we propose an algorithm to choose the best among the concerned methods while training, not after. The main idea here is that the synthesized data from the generative adversarial network (GAN) is introduced to equivalently evaluate the performance of the model trained by each continual learning method. To alleviate the potential risk of privacy invasion caused by the fake data, we randomly paired patient demographic data to the generated ECG and increased the difficulty of patient identification. In experiments, we used four different openly accessible electrocardiogram (ECG) datasets: Shaoxing and Ningbo Hospital ECG Database [12], PTB-XL [13], Georgia 12-Lead ECG Challenge Database [14], and China Physiological Signal Challenge in 2018 (CPSC 2018) [15]. Our contributions are as follows:

- We propose an algorithm to select the most suitable continual learning method in a multicenter study under a segregated environment without access to preceding datasets. To our best knowledge, this is the first approach to compare continual learning methods in advance, not ex post facto.
- Under the real-world setting involving institutions with different data distribution and data collection equipment, we validated our proposed method using four independent real-world ECG datasets.
- We utilized the fake data based on GAN to equivalently evaluate the model’s performance trained by each continual learning method. We mitigated the potential privacy risk of the fake data by randomly pairing demographic data to the generated ECG.

## Related work

### Federated learning

Federated Learning assumes that several mobile devices have privacy-sensitive data, and merging all data into single storage for training is not allowed. Each device sends the trained model to the central server, and those models are merged and distributed to each device repeatedly [16]. Federated learning can be categorized into horizontal and vertical federated learning; horizontal federated learning is introduced when datasets share the same feature space but are different in samples, and vertical federated learning is the opposite [17]. In this study, a horizontal federated learning setting is applied, assuming that the size of input data from each institution is matched.

### Continual learning

The goal of continual learning is to learn a new task while preserving pre-trained knowledge [10, 11]. However, as new tasks are added, it is inevitable to degenerate the performance of previously learned tasks. This phenomenon is called the stability-plasticity dilemma. Stability implies preserving previous knowledge, and plasticity implies integrating new knowledge [18, 19]. Continual learning methods are distinguished into three categories which are (1) replay method, (2) regularization-based method, and (3) parameter isolation method. The replay method uses samples from previous data or synthesizes fake data to train the model with current task data. The primary considerations of the replay method are how many samples to store, which representative samples to choose, and how to synthesize data to retain the previous distribution [20–22]. Meanwhile, storing sampled data may cause privacy invasion. Compared with the replay method, the regularization-based method does not require previously sampled data. Instead, it uses the additional term in the loss function to maintain the weights of essential parameters from the previous model without sampling past data [23–25]. Similarly, the parameter isolation method does not use sampled data. However, it differs from the regularization-based method because it fixes parameters allocated to each task. Thus, the number of all tasks should be defined in advance [26].

### Generative adversarial network

To equivalently evaluate the stability of continual learning, this study introduces synthesized data of generative adversarial network (GAN). GAN was first introduced by Goodfellow et al. in 2014 [27]. The GAN has two main components: generator and discriminator. The generator maps a random noise to the input space, and the discriminator classifies whether the received data is real or synthesized. Despite the brilliant idea of the GAN, it is well-known to be hard to train because no converging point exists. Wasserstein GAN (WGAN) proposed by Arjovsky et al., used the Wasserstein-1 distance defined as the distance between two different distributions [28]. With the WGAN setting, the discriminator does not classify samples as real or fake but is used to calculate Wasserstein-1 distance. To apply Wasserstein-1 distance as a differentiable loss function, however, weight clipping is necessary to maintain the discriminator to be 1-Lipschitz. To deal with weight clipping, Gulrajani et al. proposed WGAN with a gradient penalty (WGAN-GP) [29]. While most GANs focused on synthesizing images, Donahue et al. presented GAN for audio (WaveGAN) [30]. WaveGAN introduced phase shuffle operation to distract the generator from learning trivial periodic features of audio. Based on WaveGAN, Thambawita et al. proposed an electrocardiogram (ECG) synthesizer, Pulse2Pulse, which follows the overall process of WaveGAN but modifies the architecture in accordance with the structure of the standard ECG waveform data [31].

### Electrocardiogram

Electrocardiogram (ECG) is an essential test performed during a medical check and contains much information about cardiac electrical activities [32]. Throughout medical checks, clinicians can determine potential heart conditions according to the basis of ECG information. The conventional ECG is measured by multiple electrodes placed on a person’s limbs and chest. The channel of the ECG is determined according to the position of the used electrodes, and the ECG is composed of 12 channels (leads). Several studies have shown the potential of artificial intelligence-aided ECG analysis. Kwon et al. and Lin et al. showed significant performance in detecting imbalance of electrolytes, including potassium, sodium, and calcium, using ECG and deep learning methods [33, 34]. Also, Raghunath et al. used a deep neural network (DNN) to predict mortality from 12-lead ECG [35]. Kiyasseh et al. proposed CLOCS, the novel contrastive learning method for the effective representation of ECG [36]. CLOCS showed state-of-the-art performance on the downstream task, arrhythmia (abnormality in a heartbeat) detection.

## Preliminaries

### Continual learning method candidates

As the basic idea of this study is to select an appropriate continual learning method for the specific dataset, we considered three regularization-based continual learning methods as the candidates: Learning without Forgetting (LwF) [23], Elastic Weight Consolidation (EWC) [24], and Memory Aware Synapses (MAS) [25]. LwF preserves preceding knowledge by adding the knowledge distillation loss proposed by Hinton et al. to the loss function [37]. The loss function for LwF is as follows:

1 $$\begin{aligned}\mathcal{L} & \left| = \mathcal{L}_{\text{new}}\left(Y_{n},{\widehat{Y_{n}}}\right)+\lambda{*}\mathcal{L}_{\text{old}}\left(Y_{o},{\widehat{Y_{o}}}\right)\right.\\ &\left| = \mathcal{L}_{\text{new}}\left(Y_{n}, \widehat{Y_{n}}\right)+\lambda{*}\left(-\sum\limits_{i=1}^{l}{{{y}_{o}}^{{\prime\left(i\right)}}}\text{log}{{\widehat{y_o}}^{{\prime\left(i\right)}}}\right)\right.\end{aligned}$$

where *l* is the number of labels, $\lambda$ is a hyperparameter setting the importance of the old task, and ${y}_{o}{^{\prime }}^{\left(i\right)}$, $\widehat{{y}_{o}}{^{\prime }}^{\left(i\right)}$ are the knowledge distillation applied currently recorded probabilities ${y}_{o}^{\left(i\right)}$, ${\widehat{{y}_{o}}}^{\left(i\right)}$ with a hyperparameter $T$:

2 $${y}_{o}{^{\prime }}^{\left(i\right)}=\frac{{\left({y}_{o}^{\left(i\right)}\right)}^{1/T}}{{\sum\nolimits}_{j}{\left({y}_{o}^{\left(i\right)}\right)}^{1/T}}, \widehat{{y}_{o}}{^{\prime }}^{\left(i\right)}=\frac{{\left({\widehat{{y}_{o}}}^{\left(i\right)}\right)}^{1/T}}{{\sum\nolimits}_{j}{\left({\widehat{{y}_{o}}}^{\left(i\right)}\right)}^{1/T}}$$

EWC is an algorithm that retains important parameters close to their old values. To discover important parameters that contain preceding information, EWC introduces the Fisher information matrix $F$ which is approximated from the Gaussian distribution of parameters. Accordingly, the loss function for EWC is as follows:

3 $$\mathcal{L}\left(\theta \right)={\mathcal{L}}_{\mathcal{B}}\left({\theta }_{\text{new}}\right)+\lambda \sum\limits_{i}{F}_{i}{\left({\theta }_{\text{new},i}-{\theta }_{\text{old},i}\right)}^{2}$$

where ${\mathcal{L}}_{\mathcal{B}}\left({\theta }_{\text{new}}\right)$ is the loss for the new task only, and $\theta$ is the weights of the model’s parameters. Fisher information matrix is equivalent to the second derivative of the loss near a minimum and can be computed from first-order Taylor expansion of the loss, so that easy to calculate even for large models [38].

MAS also retains important parameters close to their old values, but instead of calculating gradients of the loss function, it uses the gradients of the squared ${\mathcal{l}}_{2}$ norm of output from the trained model. Thus, the importance weight ${{\Omega }}_{i}$ for parameter ${\theta }_{i}$ is:

4 $$\Omega_i=\frac1N\sum\limits_{k=1}^N\left\|\frac{\partial\left[\ell_2^2\left(M\left(x_k;\theta\right)\right)\right]}{\partial\theta_i}\right\|$$

where $M$ is trained model, ${x}_{k}$ is $k$-th data point, and $N$ is the number of data points. The loss function for MAS is equivalent to Eq. (3) by changing $F$ to ${{\Omega }}_{i}$ as follows:

5 $$\mathcal{L}\left(\theta \right)={\mathcal{L}}_{\mathcal{B}}\left({\theta }_{\text{new}}\right)+\lambda \sum\limits_{i}{{\Omega }}_{i}{\left({\theta }_{\text{new},i}-{\theta }_{\text{old},i}\right)}^{2}$$

### Arrhythmia detection model

**Fig. 1:**
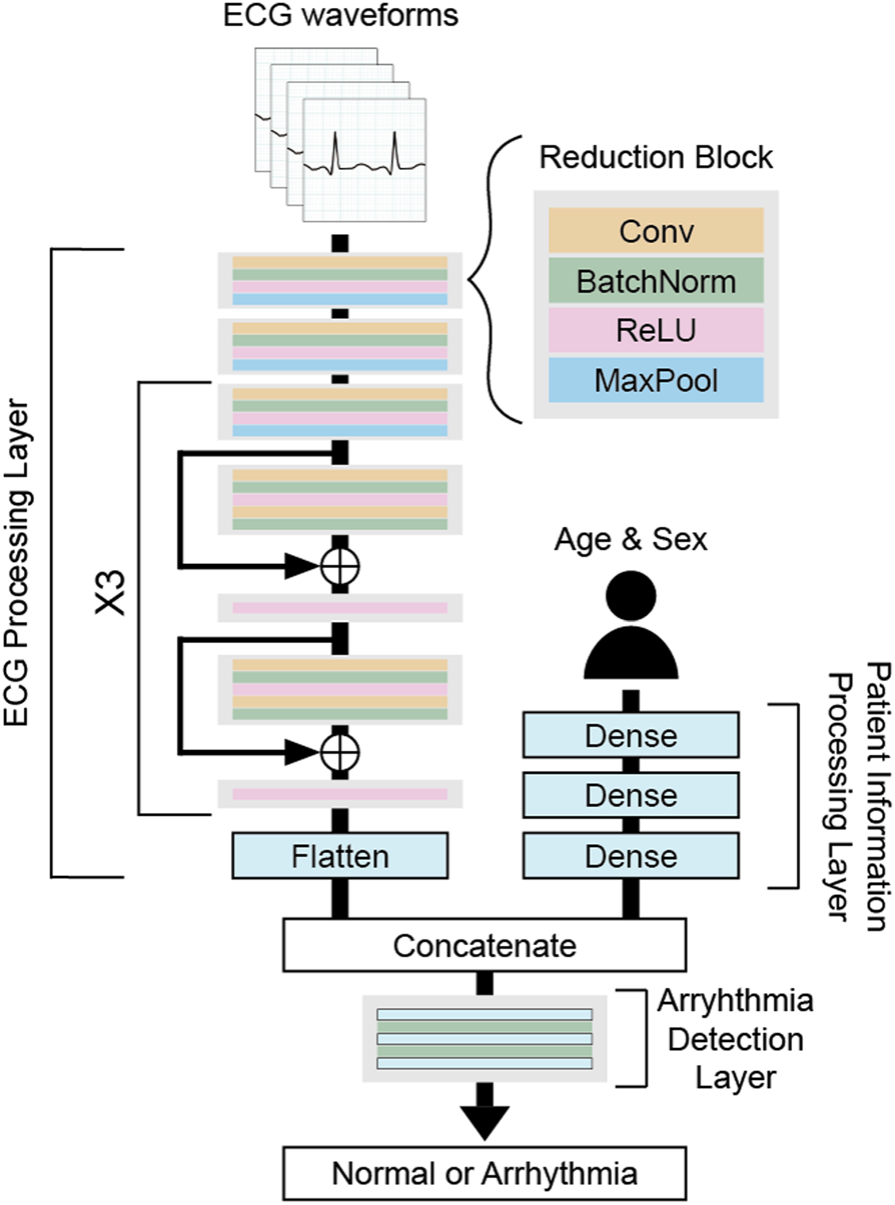
The architecture of the arrhythmia detection model. The representations of ECG waveforms and patient information including age and sex are concatenated and pass through the arrhythmia detection layer to return the probability of arrhythmia

In this study, each institution trains a model to detect arrhythmia based on ECG data, age, and sex. The model has three layers: an ECG waveform processing layer based on residual one-dimensional convolutional neural networks, a patient information processing layer that uses a multi-layer perceptron (MLP), and an arrhythmia detection layer that uses another MLP to return the probability of arrhythmia based on the concatenated outputs of the previous two layers. The model’s architecture is shown in Fig. 1, and the detailed configuration of the model is described in Table 1. Note that this architecture was used in several studies using physiological signals [35, 39]. We empirically modified the architecture for arrhythmia detection.

**Table 1:** Configuration of arrhythmia detection model. n indicates the batch size

	Kernel	Size
ECG Processing Layer		
Input		(n, 12, 5000)
Block1 (stride = 2)	(11, 12, 8 × 12 = 96)	(n, 96, 1250)
Block2 (stride = 2)	(7, 96, 96)	(n, 96, 313)
Block3 (stride = 2)	(5, 96, 96)	(n, 96, 80)
Block4 (stride = 1)	(5, 96, 96)×2	(n, 96, 80)
Block5 (stride = 1)	(5, 96, 96)×2	(n, 96, 80)
Block6 (stride = 1)	(5, 96, 16 × 12 = 192)	(n, 192, 40)
Block7 (stride = 1)	(5, 192, 192)	(n, 192, 40)
Block8 (stride = 1)	(5, 192, 192)	(n, 192, 40)
Block9 (stride = 1)	(5, 192, 32 × 12 = 384)	(n, 384, 20)
Block10 (stride = 1)	(5, 384, 384)	(n, 384, 20)
Block11 (stride = 1)	(5, 384, 384)	(n, 384, 20)
Patient Information Processing Layer		
Input		(n, 2)
Dense1	(2, 32)	(n, 32)
Dense2	(32, 64)	(n, 64)
Dense3	(64, 64)	(n, 64)
Arrhythmia Detection Layer		
Input		(n, 384 × 20 + 64 = 7744)
Dense1	(7744, 512)	(n, 512)
Dense2	(512, 256)	(n, 256)
Dense3	(256, 2)	(n, 2)

## Methods

**Fig. 2:**
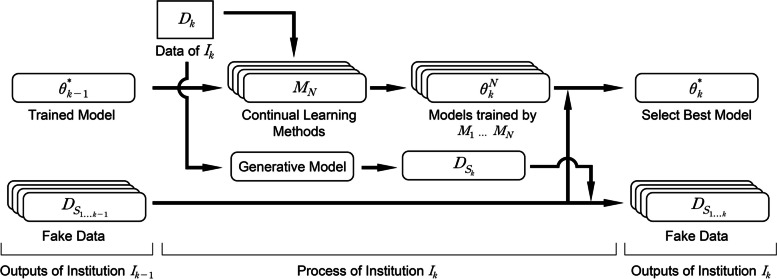
Flowchart of continual learning framework for a multicenter study

In this section, we present a continual learning framework for a multicenter study, as shown in Fig. 2. First, we present our continual learning method selection algorithm in a segregated environment without access to any previous data. Then, we describe the process of constructing fake data using a GAN-based ECG synthesizer.

### Framework notations

We represent each continual learning method as ${\text{M}}_{\text{l}}$ for $\text{l}\in \left\{1,\cdots ,\text{N}\right\}$. ${\text{D}}_{\text{k}}$ is the data of $\text{k}$-th institution. The model parameters trained by ${\text{M}}_{\text{l}}$ and ${\text{D}}_{\text{k}}$ are defined as ${{\uptheta }}_{\text{k}}^{\text{l}}$. The best-performing model’s parameters are denoted as ${{\uptheta }}_{\text{k}}^{\text{*}}$. Note that the initial state of the training model at the $\text{k}$-th institution is ${{\uptheta }}_{\text{k}-1}^{\text{*}}$. ${\text{S}}_{\text{k}}$ is the synthesizer trained by ${\text{D}}_{\text{k}}$, and ${\text{D}}_{{\text{S}}_{\text{k}}}$ is the fake data by ${\text{S}}_{\text{k}}$. For continual learning method selection, each ${{\uptheta }}_{\text{k}}^{\text{l}}$ is evaluated by the accumulated fake data $\left\{{\text{D}}_{{\text{S}}_{1}},\cdots ,{\text{D}}_{{\text{S}}_{\text{k}-1}}\right\}$. Every $\text{k}$-th institution transfers the selected model ${{\uptheta }}_{\text{k}}^{\text{*}}$, and the accumulated fake data $\left\{{\text{D}}_{{\text{S}}_{1}},\cdots ,{\text{D}}_{{\text{S}}_{\text{k}}}\right\}$ to the following institution.

### Continual learning method selection

Some studies on continual learning have succeeded and advanced the field, but they measured their performance using all the data at once after finishing all their experiments. This approach makes sense for determining the best method, but it’s not practical in real-world multicenter studies where accessing past data is not permitted. Additionally, it’s hard to guarantee that a single method will work well for all the datasets involved.

Accordingly, we assume that there is a suitable continual learning method depending on the dataset. To find the most suitable one, the stability of candidate methods should be compared under the equivalent condition without preceding data. In this study, the equivalent condition is fulfilled by the fake data, which is equally utilized to evaluate given methods, and the stability is defined as the performance of a method calculated by the fake data.

The optimal hyperparameters of continual learning methods such as ${\uplambda }$ in Eqs. (1), (3), and (5) should also be determined without preceding data. We referred to the continual hyperparameter selection introduced by De Lange et al. [10]. Our proposed continual learning method selection process in each institution is illustrated in Algorithm 1.

**Figure:**
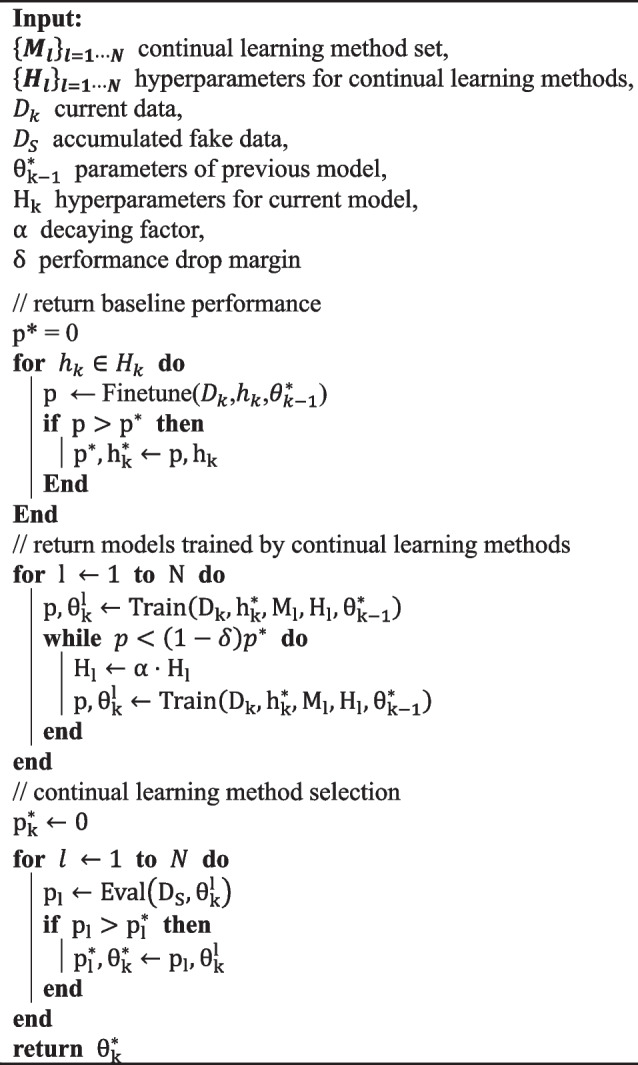
**Algorithm 1** Continual learning method selection

The proposed algorithm consists of three steps: First, the previous model ${{\uptheta }}_{\text{k}-1}^{\text{*}}$ is finetuned by ${\text{D}}_{\text{k}}$ without any regulation to preserve previous knowledge, and the baseline performance ${\text{p}}^{\text{*}}$ and the corresponding model hyperparameters ${\text{h}}_{\text{k}}^{\text{*}}$ are returned. Second, the trained model parameters ${{\uptheta }}_{\text{k}}^{\text{l}}$ for the corresponding continual learning method ${\text{M}}_{\text{l}}$ are determined. In this step, the previous model ${{\uptheta }}_{\text{k}-1}^{\text{*}}$ is trained by ${\text{M}}_{\text{l}}$. The continual hyperparameters ${\text{H}}_{\text{l}}$ are initially set to maximize stability and then alleviated by the decaying factor ${\upalpha }$ until the performance of the current data meets the reference value, which is the dropped baseline performance $\left(1-{\updelta }\right){\text{p}}^{\text{*}}$, where ${\updelta }$ is the performance drop margin. In the last step, each ${{\uptheta }}_{\text{k}}^{\text{l}}$ is evaluated by the previously accumulated fake data ${\text{D}}_{{\text{S}}_{\text{k}-1}}$. Then the best-performing model’s parameters ${{\uptheta }}_{\text{k}}^{\text{*}}$ are selected and returned.

### Fake data construction

This study mainly focused on comparing various continual learning methods in the process of multicenter study. In this context, to reflect reality, we assumed a precluded environment where access to other data is not available when only one data can be accessed for training models at one step, and we used the synthesized data as surrogate of real data for equivalent evaluation of the stability of the model trained by each continual learning method. As an ECG waveform synthesizer, Pulse2Pulse proposed by Thambawita el al., was used [31]. The original Pulse2Pulse generates an 8-channel ECG composed of lead I, II, and V1 to V6, and constructs the rest of the leads (III, aFR, aVR, aVL) by linear calculation of the eight leads. Since it was not verified whether the excluded leads (III, aFR, aVR, aVL) of all used datasets were calculated or directly measured, we modified the size of the layers of Pulse2Pulse’s generator and discriminator to synthesize the full 12-lead ECG. Keeping the original setting of Pulse2Pulse, only the sizes of the discriminator’s input layer and the generator’s input and output layers were changed from 8 to 12. The training data were separated into groups with and without arrhythmia, and the generator was trained using the data from each group. To preserve the approximate age and sex distributions of the original data, we randomly sampled data of age and sex from each group and randomly paired them to the synthesized waveforms of the corresponding group. Note that this process maintains the joint distribution of age and sex, not the complete distribution including waveforms. By this approach, we increased the difficulty of identifying individuals, ensuring that the synthesized ECG and corresponding demographic information differed significantly from the original data. Meanwhile, even though some prior studies have explored human identification using ECG [40, 41], there is currently no standardized technique for identifying individuals by ECG, and as pointed out by Thambawita et al., generating realistic synthetic data can be an alternative solution to privacy issues [31]. In this way, our fake data construction process alleviated the potential risk of privacy invasion.

### Data and code availability

All datasets used for the development and validation of the proposed framework in this study are publicly available [12–15]. The code for ECG and demographic data preprocessing, model development, and all experiments including arrhythmia detection and fake data construction is available in our source code repository at https://anonymous.4open.science/r/CLMS-FB72.

## Experiments and results

### Datasets

We conducted experiments using four publicly available ECG datasets (Shaoxing and Ningbo Hospital ECG Database, PTB-XL, Georgia 12-Lead ECG Challenge Database, and CPSC 2018) including arrhythmia labels [12–15]. Each 12-lead ECG was sampled with a frequency of 500 Hz for 10 s. All datasets contain age and sex information. In this study, we used ECGs with ages between 18 and 100. The baseline characteristics of all datasets are shown in Table 2. The ECGs were filtered by 0.5 to 40 Hz using a fifth-order bandpass Butterworth filter and scaled to range from − 1 to 1. All datasets were randomly split into training, validation, and test sets according to an 8:1:1 ratio.

**Table 2:** Baseline Characteristics of all datasets. The mean and standard deviation of age, the percentages of the male sex, and arrhythmia are presented

All Datasets	Shaoxing	PTB-XL	Georgia	CPSC
N	42,299	21,374	10,197	6696
Age	60.79 ± 16.41	59.82 ± 16.42	60.54 ± 15.4	61.18 ± 17.9
Male sex	18,445 (43.61)	10,152 (47.50)	4722 (46.31)	3092 (46.18)
Arrhythmia	34,689 (82.01)	11,996 (56.12)	8485 (83.21)	5876 (87.75)

### Training on a single domain

We first considered the effect of our continual learning framework on a single domain, as a “weak” multicenter study. Training on a single domain assumes the plain condition that the datasets are collected from each site independently having different cohort distributions, but the recording device and regional factors are shared. PTB-XL was used as a single domain and split into four non-IID (not independent and identically distributed) data because the arrhythmia of the dataset was most evenly distributed.

#### Non-IID data generation

The splitting procedure is as follows: First, divide the dataset into four groups based on age 60 and sex [42]. Second, randomly subdivide each group into ten subgroups. Then for each group, randomly select three subgroups among ten subgroups, and distribute them to the other three groups except itself. In this way, four non-IID data corresponding to each site are generated, and the summary of baseline characteristics is shown in Table 3.

**Table 3:** Baseline Characteristics of non-IID groups of PTB-XL. The mean and standard deviation of age, the percentages of the male sex, and arrhythmia are presented

PTB-XL	Site 1	Site 2	Site 3	Site 4
N	5305	4740	5705	5624
Age	51.71 ± 14.98	51.0 ± 16.38	66.48 ± 12.71	68.14 ± 13.47
Male sex	1015 (19.13)	3619 (76.35)	1015 (17.79)	4503 (80.07)
Arrhythmia	2678 (50.48)	1897 (40.98)	3875 (67.92)	3546 (63.05)

### Training on multiple domains

Non-IID data from a single domain may reflect the segregated environment to some extent. However, a real-world multicenter study consists of more different datasets in the aspect of the cohort distribution, the data collecting devices, the structure of databases, and other hardly explainable regional factors such as overall income level, ethnicity, and climate. Accordingly, we set the experiment on multiple domains as a “strong” multicenter study, using four independently different datasets, PTB-XL ECG dataset (PTB-XL), Shaoxing and Ningbo Hospital ECG Database (Shaoxing), Georgia 12-Lead ECG Challenge Database (Georgia), and CPSC2018 dataset (CPSC). As shown in Table 1, PTB-XL has a ratio of arrhythmia much different from the rest datasets. Regarding data devices, the PTB-XL dataset was recorded by devices from the Schiller AG, while the Shaoxing dataset was recorded by the GE MUSE ECG system. As to region, Georgia was collected in the USA, while Shaoxing and CPSC were collected in China.

### Experimental details

#### Supervised learning

For the baseline of supervised learning, we trained the arrhythmia detection model using individual data from each site and all merged data. Cross entropy loss was to be minimized considering the class imbalance of the training set as follows:

$$\ell\left(\text{x},\text{y}\right)=\sum\limits_{\text{i}=1}^{\text{N}}\frac{\text{N}}{\sum\nolimits_{\text{j}=1}^{\text{N}}1\left\{{\text{y}}_{\text{j}}={\text{y}}_{\text{i}}\right\}}\ {\text{l}}_{\text{i}}$$

where $\text{N}$ is the number of training data, ${\text{l}}_{\text{i}}$ is the loss of $\text{i}$-th data, and ${\text{y}}_{\text{i}}$ is the label of $\text{i}$-th data. The training epoch was set to 100, and the model with the best validation area under the receiver operating characteristic curve (AUROC) was selected. The model was optimized by Adam [43], with a learning rate of 0.0001, and the batch size was set to 256.

#### Federated learning

FedAvg by McMahan et al. was adopted as a baseline of federated learning [16]. The parameter averaging process is as follows:

$${\text{w}}_{\text{t}}\leftarrow \sum\limits_{\text{k}=1}^{\text{K}}\frac{{\text{n}}_{\text{k}}}{\text{nw}}_{\text{t}}^{\text{k}}$$

where ${\text{w}}_{\text{t}}$ denotes the merged data, ${\text{w}}_{\text{t}}^{\text{k}}$ the updated parameter of $\text{k}$-th institution, $\text{K}$ the number of involved institutions, $\text{n}$ the total number of data, ${\text{n}}_{\text{k}}$ the number of data of $\text{k}$-th institution. We additionally conducted federated learning experiments with FedProx by Li et al. [44], which adds an L2 regularization term to the loss function $\text{L}$ in the local training of FedAvg as follows [45]:

$$\stackrel{\sim}{\text{L}}\leftarrow \text{L} + \frac{\mu }{2} \parallel {\text{w}}_{\text{t}}^{\text{k}}-{\text{w}}_{\text{t}}{\parallel }_{2}^{2}$$

where $\mu$ is a hyperparameter that controls the regularization.

For every round, each site trained the model according to the manner of supervised learning with 20 epochs, and the training was early stopped if there was no increase of the AUROC for more than five epochs. This process was repeated for 30 rounds, and the model with the best weighted average AUROC was selected. The formula for weighted average AUROC in this study is as follows:

$$\text{Weighted average AUROC}=\sum\limits_{\text{i}}\frac{{\text{N}}_{\text{i}}}{\text{N}}\times {\text{AUROC}}_{\text{i}}$$

where ${\text{N}}_{\text{i}}$ denotes the number of data in the $\text{i}$-th institution, $\text{N}$ the total number of data across all institutions.

#### Finetuning and continual learning

For finetuning and continual learning, the order of the sites should be considered. However, since there is no standard ordering technique [10], we arbitrarily determined training order by sorting from small to large and vice versa based on each site’s dataset size (N in Tables 1 and 2). For each site, the training epoch was set to 100, and the training process was early stopped if there was no increase in the AUROC for more than ten epochs. No regularization was applied to finetuning. Continual learning followed Algorithm 1, and LwF, EWC, and MAS were used as method candidates. For the hyperparameter of each method, ${\uplambda }$ in Eqs. (1), (3) and (5) was used and initially set to 1, and $\text{T}$ in Eq. (2) was set to 10, empirically. The decaying factor ${\upalpha }$ and performance drop margin ${\updelta }$ were also empirically set to 0.9 and 0.95, respectively.

#### Generative model training

**Fig. 3:**
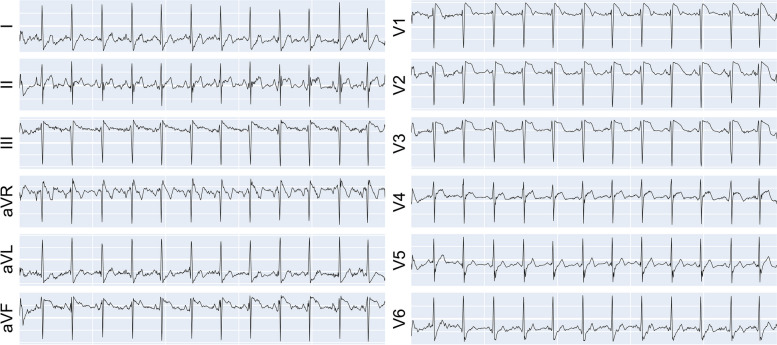
A sample of synthesized normal 12-lead ECG from PTB-XL

To evaluate continual learning methods, we used synthesized data from a generative model. As a generative model, we adopted the modified Pulse2Pulse to synthesize 12-lead ECGs. The training epoch was set to 2000, and the early stopping was activated if there was no decrease of the negative critic loss of WGAN-GP for more than 100 epochs [29]. The model was optimized by Adam [43], with a learning rate of 0.0001, and the batch size was set to 64. For the ECG synthesizer, the generator was trained once while the discriminator was trained for five epochs. The parameters of the first trained synthesizer were transferred to the following site, and the parameters were used to initialize the new synthesizer to reduce the training time. Note that we only transferred the trained synthesizer with no previously sampled data. A sample of synthesized normal ECG is shown in Fig. 3. The comparison of ECG features extracted from the original and synthesized ECG is shown in Supplementary Table 1, confirming that our generator successfully addressed mode collapse [46].

#### Computational information

The total numbers of parameters and the floating-point operations (FLOPs) of the arrhythmia detection model, ECG generator, and ECG discriminator were 6,631,234, 10,634,969, and 203,923,151 and 0.211G, 0.321G, and 6.385G FLOPs, respectively. We utilized 2 AMD EPYC 7763 CPUs and 1 NVIDIA RTX A6000 GPU for our implementation. For the arrhythmia detection task, we allocated 5473 MB GPU memory with a batch size of 256 for training and achieved a training time of approximately 0.08 s per batch. During the ECG generating task, we allocated 21,569 MB GPU memory with a batch size of 64 and obtained a training time of 2.54 s per batch.

### Experimental results

#### Performance on a single domain

**Fig. 4:**
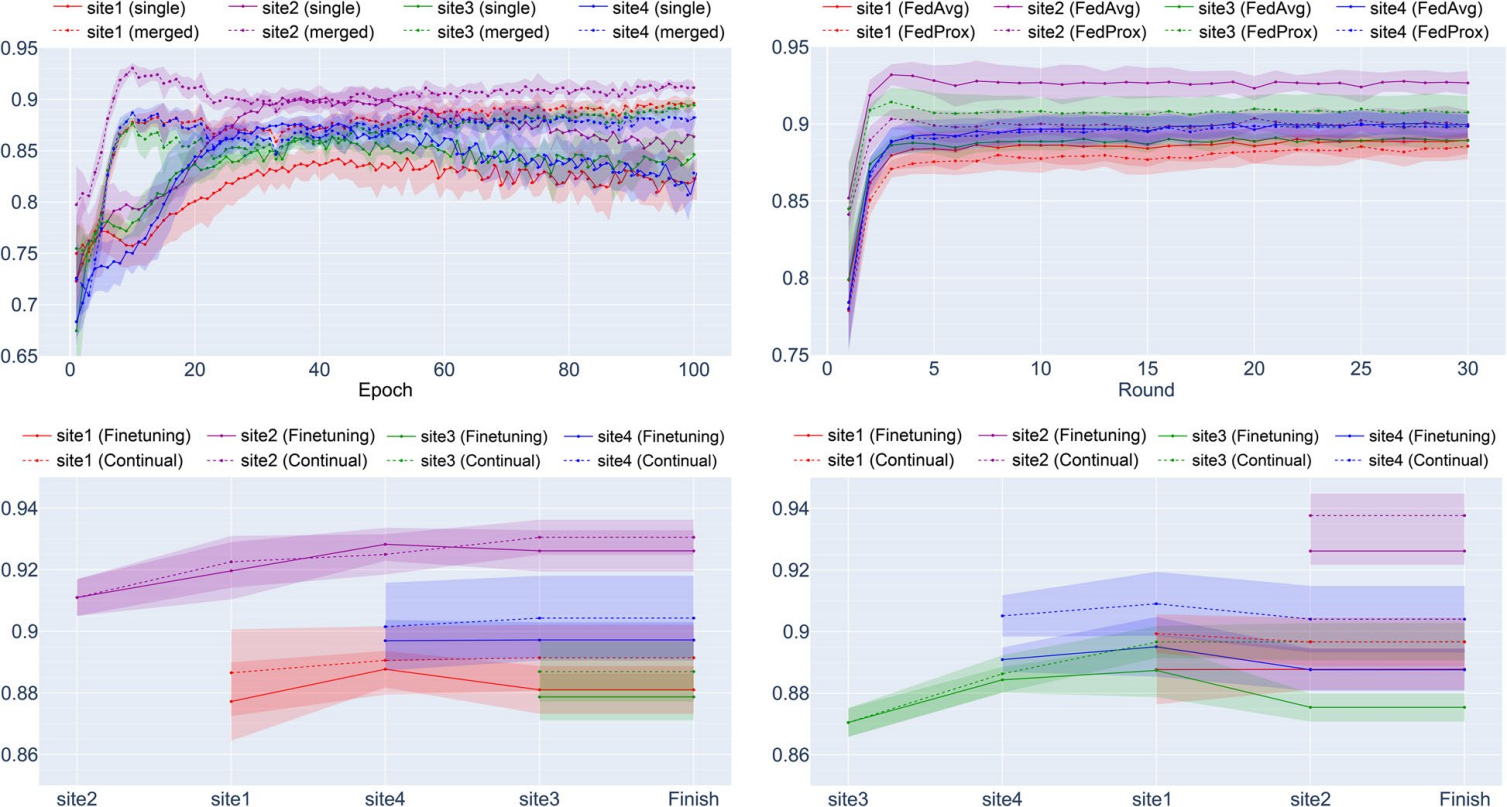
Validation AUROC of all methods on a single domain. (upper left) supervised learning, (upper right) federated learning, (lower left) continual learning (small to large), (lower right) continual learning (large to small). Results are averaged across five random seeds and the shaded part indicates one standard deviation

The test performances of all methods on a single domain are presented in Table 4. Note that the overall AUROC was calculated by the weighted average AUROC described in Federated Learning section. We trained the model using all merged data assuming full accessibility, the performance of supervised learning for merged data showed consistently better performance than single supervised learning. The overall performance was best in our proposed framework with large-to-small order, resulting in an AUROC = 0.914, but since the data from all sites are derived from the identical dataset, PTB-XL, the performance difference between the methods was hypothesized not to be very large. Figure 4 shows the fluctuation of validation AUROC of all methods as the training process goes on. For continual learning method selection, the selection rates of LwF, EWC, and MAS throughout all experiments were 10.0%, 40.0%, and 50.0%, respectively.

**Table 4:** Test performances of all methods on a single domain (PTB-XL) are presented. The mean and standard deviation across five random seeds are shown. Bold reflects the method with the best performance. The overall performance is the weighted average AUROC by the number of data in each site. Bold is the best and underlined is the second best

	**Site 1**	**Site 2**	**Site 3**	Site 4	Overall
Supervised (baseline)					
Single data	0.874 ± 0.010	0.902 ± 0.013	0.890 ± 0.003	0.894 ± 0.009	
Merged data	0.903 ± 0.013	0.935 ± 0.010	0.910 ± 0.009	0.911 ± 0.008	0.914 ± 0.010
Federated					
FedAvg	0.901 ± 0.003	0.917 ± 0.007	0.909 ± 0.006	**0.915 ± 0.006**	0.910 ± 0.005
FedProx	0.902 ± 0.003	0.925 ± 0.004	0.906 ± 0.008	0.906 ± 0.004	0.909 ± 0.003
Finetuning					
Small to Large	0.899 ± 0.006	0.918 ± 0.003	0.903 ± 0.005	0.905 ± 0.004	0.906 ± 0.003
Large to Small	0.889 ± 0.005	0.926 ± 0.005	0.898 ± 0.006	0.903 ± 0.004	0.903 ± 0.004
Continual					
Small to Large	0.894 ± 0.010	0.923 ± 0.009	0.907 ± 0.010	0.904 ± 0.010	0.906 ± 0.009
Large to Small	**0.903 ± 0.006**	**0.929 ± 0.011**	**0.916 ± 0.007**	0.909 ± 0.006	**0.914 ± 0.007**

#### Performance on multiple domains

**Fig. 5:**
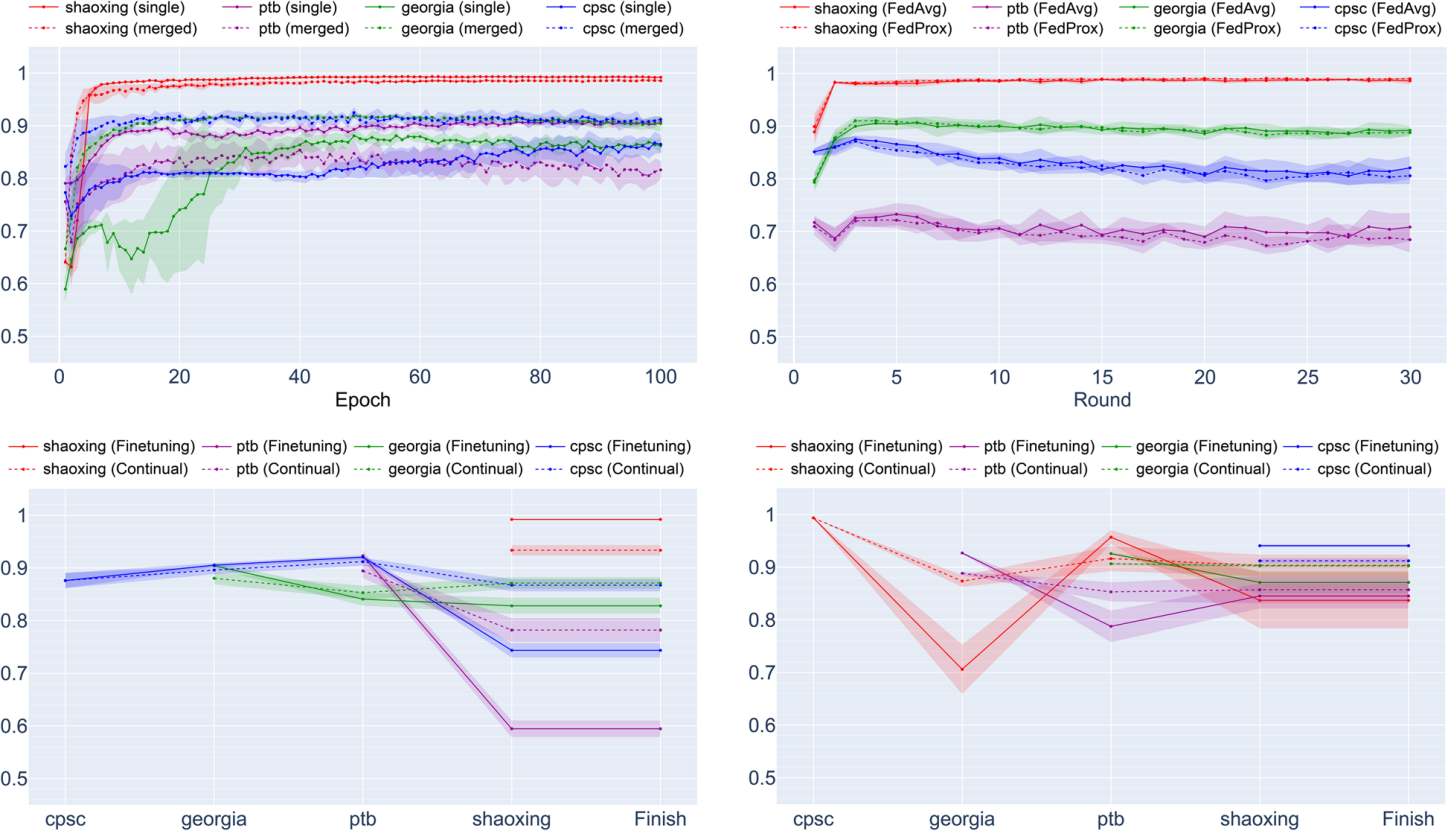
Validation AUROC of all methods on multiple domains. (upper left) supervised learning, (upper right) federated learning, (lower left) continual learning (small to large), (lower right) continual learning (large to small). Results are averaged across five random seeds and the shaded part indicates one standard deviation

In Table 5, the test performances of all methods on multiple domains are presented. In supervised learning, the performance were improved by training with the merged data including each other, but the performance on PTB-XL, which has a significantly different arrhythmia ratio compared to the other datasets, became worse (AUROC 0.930 $\to$ 0.842). FedAvg and FedProx also showed weak performance on PTB-XL with an AUROC of 0.751 and 0.735, while the overall performance was best with an AUROC of 0.901 and 0.900, respectively. Finetuning with small-to-large order showed the best performance for the Shaoxing dataset; however, this is because the model was trained by the Shaoxing dataset at last in this order. The result of the large-to-small order showed the best performance in CPSC2018 because of the same reason. Among all methods, only continual learning with large-to-small order achieved AUROC over 0.87 on all datasets, with an overall AUROC = 0.897, which is only 0.004 lower than the best score (FedAvg). The results of the small-to-large order showed relatively weak performance than the large-to-small order like finetuning, but the decline in performance during the training was much smaller than finetuning. For all orders, continual learning maintained the performance of each site as training progressed by suppressing catastrophic forgetting, as shown in Fig. 5. For continual learning method selection, the selection rates of LwF, EWC, and MAS throughout all experiments were 53.3%, 16.7%, and 30.0%, respectively.

**Table 5:** Test performances of all methods on multiple domains are presented. The mean and standard deviation across five random seeds are shown. The overall performance is the weighted average AUROC by the number of data in each site. Bold is the best and underlined is the second best

	Shaoxing	PTB-XL	Georgia	CPSC	Overall
Supervised (baseline)					
Single data	0.994 ± 0.001	0.930 ± 0.003	0.874 ± 0.008	0.867 ± 0.019	
Merged data	0.977 ± 0.003	0.842 ± 0.023	0.916 ± 0.002	0.924 ± 0.007	0.929 ± 0.005
Federated					
FedAvg	0.980 ± 0.004	0.751 ± 0.013	0.901 ± 0.007	0.876 ± 0.013	**0.901 ± 0.003**
FedProx	0.984 ± 0.001	0.735 ± 0.010	**0.906 ± 0.004**	0.882 ± 0.007	0.900 ± 0.003
Finetuning					
Small to Large	**0.994 ± 0.000**	0.584 ± 0.017	0.829 ± 0.013	0.768 ± 0.017	0.845 ± 0.007
Large to Small	0.839 ± 0.055	0.856 ± 0.024	0.874 ± 0.023	**0.939 ± 0.003**	0.856 ± 0.027
Continual					
Small to Large	0.935 ± 0.010	0.785 ± 0.023	0.876 ± 0.012	0.871 ± 0.013	0.883 ± 0.006
Large to Small	0.908 ± 0.020	**0.873 ± 0.026**	0.896 ± 0.004	0.912 ± 0.007	0.897 ± 0.005

## Discussion

In this study, we proposed a continual learning framework for a multicenter study with a segregated environment where data sharing is strictly prohibited. We focused on evaluating various continual learning methods without preceding data during the training, not after. The fake data was synthesized from a proper generative model to evaluate each continual learning method equivalently, and in this process, no raw data was shared. To mitigate the potential risk of privacy invasion, we randomly paired the sampled demographic data to the synthesized waveform, disturbing the original data distribution. Our proposed framework with proper order (large-to-small) showed competitive performance (AUROC 0.897) compared to federated learning (AUROC 0.901) and successfully suppressed catastrophic forgetting regardless of the dataset. Beyond the performance, our framework has higher utility than federated learning since continual learning does not require a central server which is an essential component of federated learning. The selection rates of method candidates (LwF, EWC, MAS) were not one-sided, and this verifies our assumption that a single method is not omnipotent for all involved datasets in a real-world setting and there could be a proper method to be selected for specific data.

There are several future works to improve the study. We proposed the continual learning method selection process based on the fake data by a generative model. However, discovering and training a proper generative model for specific kinds of data requires a lot of effort. Towards the goal of equivalent evaluation of continual learning methods, considering more efficient and mathematically reasonable evaluation processes would be a future work. Continual learning methods usually have the additional term in loss function to control catastrophic forgetting. Thus, directly comparing these terms in a coordinated scale would reduce the evaluation time without a generative model. On the other hand, there could be persistent privacy risks despite utilizing fake data, and thus, we plan to evaluate privacy invasion through reasonable metrics for privacy-preserved fake data. Also, we performed experiments with orders from small to large and vice versa to set the order according to the criteria (despite weak significance) instead of random ordering, because there is no standard ordering technique yet [10]. However, this approach might not be feasible if there are many sites or the sample size in each domain is similar or dynamic, and the other ordering techniques are to be further explored. Meanwhile, this study was performed with standard 12-lead ECGs with a frequency of 500 Hz for 10 s, so the methods of the study should be validated by another format of ECG, such as Holter monitor records, overcoming diverse measurement environments simultaneously. Lastly, expanding continual learning method candidates and analyzing the impact of our framework on the other models, such as lightweight deep learning models, remains future work.

### Supplementary Information

**Supplementary material 1.**

## Data Availability

All data used in this study is publicly available. The source code repository is available in https://github.com/kicarussays/CLMS.

## References

[1] Rieke N, et al. The future of digital health with federated learning. Npj Digit Med. 2020;3(1):119. 10.1038/s41746-020-00323-1. 33015372 10.1038/s41746-020-00323-1 PMC7490367

[2] Kaissis GA, et al. Secure, privacy-preserving and federated machine learning in medical imaging. Nat Mach Intell. 2020;2(6):305–11. 10.1038/s42256-020-0186-1. 10.1038/s42256-020-0186-1

[3] Nguyen DC, et al. Federated learning for smart healthcare: a survey. ACM Comput Surv (CSUR). 2022;55(3):1–37. 10.1145/3501296. 10.1145/3501296

[4] Liu X, et al. Federated Neural Architecture Search for Medical Data Security. IEEE Trans Industr Inf. 2022;18(8):5628–36. 10.1109/TII.2022.3144016. 10.1109/TII.2022.3144016

[5] Sarma KV, et al. Federated learning improves site performance in multicenter deep learning without data sharing. J Am Med Inform Assoc. 2021;28(6):1259–64. 10.1093/jamia/ocaa341. 33537772 10.1093/jamia/ocaa341 PMC8200268

[6] Ye D, et al. Federated Learning in Vehicular Edge Computing: a selective Model Aggregation Approach. IEEE Access. 2020;8:23920–35. 10.1109/ACCESS.2020.2968399. 10.1109/ACCESS.2020.2968399

[7] Wang KIK, et al. Federated Transfer learning based Cross-domain Prediction for Smart Manufacturing. IEEE Trans Industr Inf. 2022;18(6):4088–96. 10.1109/TII.2021.3088057. 10.1109/TII.2021.3088057

[8] Cui X, Lu S, Kingsbury B. Federated Acoustic Modeling for Automatic Speech Recognition. in ICASSP 2021–2021 IEEE International Conference on Acoustics, Speech and Signal Processing (ICASSP). 2021. 10.1109/icassp39728.2021.9414207 PMC10286626 37351441

[9] Zhang C, et al. A survey on federated learning. Knowl Based Syst. 2021;216:106775. 10.1016/j.knosys.2021.106775. 10.1016/j.knosys.2021.106775

[10] Lange MD, et al. A continual learning survey: defying forgetting in classification tasks. IEEE Trans Pattern Anal Mach Intell. 2022;44(7):3366–85. 10.1109/TPAMI.2021.3057446. 33544669 10.1109/TPAMI.2021.3057446

[11] Chen Z, Liu B. Lifelong machine learning. Synthesis lectures on Artificial Intelligence and Machine Learning. 2018. 12(3):1–207.

[12] Zheng J, et al. A 12-lead electrocardiogram database for arrhythmia research covering more than 10,000 patients. Sci Data. 2020;7(1):48. 10.1038/s41597-020-0386-x. 32051412 10.1038/s41597-020-0386-x PMC7016169

[13] Wagner P, et al. Sci Data. 2020;7(1):154. 10.1038/s41597-020-0495-6. PTB-XL, a large publicly available electrocardiography dataset. 10.1038/s41597-020-0495-6 PMC7248071 32451379

[14] Perez Alday EA, et al. Classification of 12-lead ECGs: the PhysioNet/Computing in Cardiology Challenge 2020. Physiol Meas. 2020;41(12):124003. 10.1088/1361-6579/abc960. 10.1088/1361-6579/abc960 PMC8015789 33176294

[15] Liu F, et al. An open access database for evaluating the algorithms of electrocardiogram rhythm and morphology abnormality detection. J Med Imaging Health Inf. 2018;8(7):1368–73. 10.1166/jmihi.2018.2442. 10.1166/jmihi.2018.2442

[16] McMahan B, et al. Communication-efficient learning of deep networks from decentralized data. Artificial intelligence and statistics. PMLR; 2017.

[17] Yang Q, et al. Federated Machine Learning: Concept and Applications. ACM Trans Intell Syst Technol. 2019;10(2).): p. Article 12 10.1145/3298981.

[18] Mermillod M, Bugaiska A, BONIN P. The stability-plasticity dilemma: investigating the continuum from catastrophic forgetting to age-limited learning effects. Front Psychol. 2013;4. 10.3389/fpsyg.2013.00504. 10.3389/fpsyg.2013.00504 PMC3732997 23935590

[19] Grossberg S. Nonlinear neural networks: principles, mechanisms, and architectures. Neural Netw. 1988;1(1):17–61. 10.1016/0893-6080(88)90021-4. 10.1016/0893-6080(88)90021-4

[20] Lopez-Paz D, Ranzato MA. Gradient episodic memory for continual learning. Adv Neural Inf Process Syst. 2017;30.

[21] Rebuffi S-A et al. icarl: Incremental classifier and representation learning. in Proceedings of the IEEE conference on Computer Vision and Pattern Recognition. 2017.

[22] Shin H et al. Continual learning with deep generative replay. Adv Neural Inf Process Syst. 2017;30.

[23] Li Z, Hoiem D. Learning without forgetting. IEEE Trans Pattern Anal Mach Intell. 2018;40(12):2935–47. 10.1109/TPAMI.2017.2773081. 29990101 10.1109/TPAMI.2017.2773081

[24] Kirkpatrick J, et al. Overcoming catastrophic forgetting in neural networks. Proc Natl Acad Sci. 2017;114(13):3521–6. 10.1073/pnas.1611835114. 28292907 10.1073/pnas.1611835114 PMC5380101

[25] Aljundi R et al. Memory aware synapses: Learning what (not) to forget. in Proceedings of the European conference on computer vision (ECCV). 2018.

[26] Mallya A, Lazebnik S, Packnet. Adding multiple tasks to a single network by iterative pruning. in Proceedings of the IEEE conference on Computer Vision and Pattern Recognition. 2018.

[27] Goodfellow I, et al. Generative adversarial networks. Commun ACM. 2020;63(11):139–44. 10.1145/3422622. 10.1145/3422622

[28] Arjovsky M, Chintala S, Bottou L, Wasserstein Generative Adversarial Networks, in Proceedings of the 34th International Conference on Machine Learning, Doina P, Yee Whye T. Editors. 2017, PMLR: Proceedings of Machine Learning Research. p. 214–223.

[29] Gulrajani I et al. Improved training of Wasserstein Gans. Adv Neural Inf Process Syst. 2017;30.

[30] Donahue C, McAuley J, Puckette M. Adversarial audio synthesis. arXiv Preprint arXiv:1802 04208. 2018. 10.48550/arXiv.1802.04208. 10.48550/arXiv.1802.04208

[31] Thambawita V, et al. DeepFake electrocardiograms using generative adversarial networks are the beginning of the end for privacy issues in medicine. Sci Rep. 2021;11(1):21896. 10.1038/s41598-021-01295-2. 34753975 10.1038/s41598-021-01295-2 PMC8578227

[32] Ghanem RN, et al. Heart-surface reconstruction and ECG electrodes localization using fluoroscopy, epipolar geometry and stereovision: application to noninvasive imaging of cardiac electrical activity. IEEE Trans Med Imaging. 2003;22(10):1307–18. 10.1109/TMI.2003.818263. 14552584 10.1109/TMI.2003.818263 PMC2034496

[33] Kwon J-m, et al. A deep learning algorithm to detect anaemia with ECGs: a retrospective, multicentre study. Lancet Digit Health. 2020;2(7):e358-e367. 10.1016/S2589-7500(20)30108-4. 10.1016/S2589-7500(20)30108-4 33328095

[34] Lin C, et al. Point-of-care artificial intelligence-enabled ECG for dyskalemia: a retrospective cohort analysis for accuracy and outcome prediction. Npj Digit Med. 2022;5(1):8. 10.1038/s41746-021-00550-0. 35046489 10.1038/s41746-021-00550-0 PMC8770475

[35] Raghunath S, et al. Deep neural networks can predict new-onset atrial fibrillation from the 12-lead ECG and help identify those at risk of atrial fibrillation–related stroke. Circulation. 2021;143(13):1287–98. 10.1161/CIRCULATIONAHA.120.047829. 10.1161/CIRCULATIONAHA.120.047829 PMC7996054 33588584

[36] Kiyasseh D, Zhu T, Clifton DA. CLOCS: Contrastive Learning of Cardiac Signals Across Space, Time, and Patients, in Proceedings of the 38th International Conference on Machine Learning, M. Marina and Z. Tong, Editors. 2021, PMLR: Proceedings of Machine Learning Research. p. 5606–5615.

[37] Hinton G, Vinyals O, Dean J. Distilling the knowledge in a neural network. arXiv Preprint arXiv:1503 02531. 2015. 10.48550/arXiv.1503.02531. 10.48550/arXiv.1503.02531

[38] Pascanu R, Bengio Y. Revisiting natural gradient for deep networks. arXiv Preprint arXiv:1301 3584. 2013. 10.48550/arXiv.1301.3584. 10.48550/arXiv.1301.3584

[39] Kwon Jm, et al. Artificial intelligence for detecting electrolyte imbalance using electrocardiography. Ann Noninvasive Electrocardiol. 2021;26(3):e12839. 10.1111/anec.12839. 33719135 10.1111/anec.12839 PMC8164149

[40] Kim B-H, Pyun J-Y. Identification for personal authentication using LSTM-Based deep recurrent neural networks. Sensors. 2020;20. 10.3390/s20113069. 10.3390/s20113069 PMC7309053 32485827

[41] Li Y, et al. Toward improving ECG biometric identification using cascaded convolutional neural networks. Neurocomputing. 2020;391:83–95. 10.1016/j.neucom.2020.01.019. 10.1016/j.neucom.2020.01.019

[42] Mirza M, et al. Mechanisms of arrhythmias and conduction disorders in older adults. Clin Geriatr Med. 2012;28(4):555–73. 10.1016/j.cger.2012.08.005. 23101571 10.1016/j.cger.2012.08.005 PMC3610528

[43] Kingma DP, Ba J. Adam: A method for stochastic optimization. arXiv preprint arXiv:1412.6980, 2014 10.48550/arXiv.1412.6980.

[44] Li T, et al. Federated optimization in heterogeneous networks. Proc Mach Learn Syst. 2020;2:429–50.

[45] Hwang H et al. Towards the Practical Utility of Federated Learning in the Medical Domain, in Proceedings of the Conference on Health, Inference, and Learning, J.M. Bobak, Editors. 2023, PMLR: Proceedings of Machine Learning Research. p. 163–181.

[46] Sheta A, et al. Diagnosis of obstructive sleep apnea from ECG signals using machine learning and deep learning classifiers. Appl Sci. 2021;11. 10.3390/app11146622.

