# Supplementary material for: Continual learning framework for a multicenter study with an application to electrocardiogram
Source: BMC Med Inform Decis Mak. 2024 Mar 6;24:67. doi: 10.1186/s12911-024-02464-9 (PMC11331660; doi:10.1186/s12911-024-02464-9)
Supplement: Supplementary file 1 — Supplementary material 1. [file 12911_2024_2464_MOESM1_ESM.docx]

**Supplementary Table 1.** Comparison of ECG features extracted from original and synthesized ECGs. The distributions of original and synthesized data are similar. This finding supports the conclusion that our generator successfully addressed the mode collapse.

| **ECG Features** | Shaoxing |  | PTB-XL |  | Georgia |  | CPSC |  |
| --- | --- | --- | --- | --- | --- | --- | --- | --- |
|  | Original | Synthesized | Original | Synthesized | Original | Synthesized | Original | Synthesized |
| P wave duration | 0.067±0.028 | 0.070±0.026 | 0.080±0.023 | 0.074±0.019 | 0.069±0.023 | 0.062±0.021 | 0.066±0.023 | 0.069±0.018 |
| PR interval | 0.136±0.039 | 0.140±0.030 | 0.147±0.035 | 0.149±0.026 | 0.141±0.039 | 0.144±0.030 | 0.137±0.037 | 0.145±0.026 |
| PP interval | 0.845±0.247 | 0.798±0.220 | 0.842±0.166 | 0.816±0.150 | 0.807±0.199 | 0.775±0.179 | 0.779±0.170 | 0.730±0.131 |
| PR segment | 0.069±0.041 | 0.071±0.030 | 0.067±0.037 | 0.075±0.027 | 0.073±0.040 | 0.082±0.030 | 0.071±0.039 | 0.075±0.025 |
| QRS duration | 0.109±0.048 | 0.099±0.037 | 0.106±0.043 | 0.096±0.023 | 0.100±0.039 | 0.093±0.027 | 0.102±0.038 | 0.093±0.016 |
| QT duration | 0.340±0.089 | 0.314±0.088 | 0.351±0.070 | 0.321±0.071 | 0.338±0.084 | 0.303±0.076 | 0.329±0.072 | 0.296±0.059 |
| RR interval | 0.845±0.247 | 0.798±0.221 | 0.842±0.166 | 0.815±0.150 | 0.806±0.199 | 0.775±0.178 | 0.778±0.170 | 0.729±0.131 |
| ST segment | 0.130±0.063 | 0.128±0.051 | 0.137±0.061 | 0.141±0.053 | 0.140±0.065 | 0.135±0.057 | 0.134±0.059 | 0.118±0.042 |
| ST-T segment | 0.231±0.074 | 0.215±0.068 | 0.245±0.063 | 0.225±0.063 | 0.238±0.076 | 0.210±0.066 | 0.227±0.065 | 0.203±0.055 |
| TP interval | 0.371±0.170 | 0.343±0.150 | 0.345±0.133 | 0.346±0.116 | 0.328±0.152 | 0.328±0.135 | 0.312±0.130 | 0.287±0.098 |
